# Commentary: Cortical Plasticity and Olfactory Function in Early Blindness

**DOI:** 10.3389/fnhum.2016.00689

**Published:** 2017-01-09

**Authors:** Alessandra Fiore, Mariella Pazzaglia

**Affiliations:** ^1^Department of Psychology, University of Rome “La Sapienza”Rome, Italy; ^2^IRCCS Santa Lucia FoundationRome, Italy

**Keywords:** olfactory, visual, plasticity, multimodal integration, rehabilitation, motor activity

Humans beings are visual creatures, which means that they preferentially map the world guided largely by sight (Aglioti and Pazzaglia, 2010). However, multiple senses contribute to the creation of an integrated experience of the real world (Aglioti and Pazzaglia, 2011). Among these, the olfactory sense is undoubtedly the most neglected. One of the reasons for this is that odors do not always prevail at a conscious level (Sobel et al., 1999), even though olfactory receptors are continuously active in order to acquire sensory, motor and affective information (Pazzaglia, 2015). A second reason is the fact that sight occasionally prevails over, or even hides, the information provided by other senses. In a recent stimulating and timely article, Araneda and colleagues presented an important view of the pathophysiological mechanisms of plasticity that occur in the visual brain of early blind patients (Araneda et al., 2016). The authors produced a behavioral and anatomo-physiological perspective on the guiding role of olfactory information when sight is lacking. Behaviorally, early blind subjects perform better than sighted individuals when it comes to odor detection and awareness, indicating that the sense of smell is highly plastic and suggesting the possibility of incremental odor processing by way of learning and experience. Neurally, active olfactory processing occurs within the occipital cortex, suggesting the privileged access of odor to other, seemingly unrelated, sensory systems and neural regions.

The study of Araneda and colleagues also provides an excellent opportunity to investigate olfactory information, not only in the complete absence of visual mediation (e.g., in congenitally blind individuals), but also in terms of understanding the value of this extraordinary plasticity in relation to the enhanced olfactory abilities of many of our sensations, perceptions, and higher-order cognitive processing. This is an important topic, and the contribution of cross-modal visual-olfactory interaction can certainly be emphasized, as this is supported by much experimental evidence in terms of highlighting the implications in the field of clinical studies. The congruent odor of an object facilities how it is visually represented (Verhagen and Engelen, 2006), and the concurrent presence of the visual cues of a given object may also enhance olfactory performances (Gottfried and Dolan, 2003). The relevance of olfaction in visual areas has been demonstrated in a causative study using repetitive transcranial magnetic stimulation, which showed that olfactory performances improve when occipital areas in the brain are stimulated, even in seeing humans (Jadauji et al., 2012). The merging of a visual representation enables healthy individuals to optimize their identification and discrimination of an object based on smell alone. Analogously, neuroimaging studies have reported modulation in visual cortices during purely olfactory performances (Qureshy et al., 2000; Zatorre et al., 2000; Dade et al., 2002), hinting at genuine cross-modal neural modulation evoked by olfactory cues. In addition to conveying visual information, it is known that odors also trigger more dynamic information about visual motor states (Kuang and Zhang, 2014).

The merging of visual and olfactory information facilitates the kinematics of reach-to-grasp movements and understanding actions (Tubaldi et al., 2011). When a person observes someone else grasping an object, the neurons that control the hand muscles in the observer become more alert, as if the individual is also preparing to perform the same action (Fadiga et al., 1995). If someone smells the aroma of a strawberry while simultaneously observing someone else grasping this fruit, the olfactory information activates in the observer the brain regions involved in the congruent body movements (Rossi et al., 2008). This facilitation effect of olfaction on movement parameters is specific to the muscles that guide the observed movement (Castiello, 1997). Conversely, the perception of smelling a different food (orange) object did not induce any facilitation of the corticospinal system.

Clinically, being driven by visual-olfactory inputs to the mapping of actions could assuage motor disturbances. In Parkinson's disease (PD), for example (Castiello et al., 2009; Parma et al., 2013a), patients present, among other symptoms, with an impairment of the kinematics of movements. Like healthy subjects, a clear facilitation with the kinematics of reaching and grasping movements was found in patients with PD in conditions of coherent actions between the object and odor. This suggests that cross-modal processing optimizes, not only perceptive, but also motor, functioning. Nevertheless, in clinical applications, the imitation of reach-to-grasp movements has been facilitated in a condition paired with the smell of the subject's own mother in Autism Spectrum Disorder (ASD) (Parma et al., 2013b). The smell of a stranger did not, however, modulate automatic visual-motor actions. Accordingly, a familiar body odor may promote socio-cognitive behavior such as the spontaneous imitation that is impaired in ASD. Together, these studies show that the olfactory modality may interact with visual-motor behavior in clinical and healthy populations in order to integrate perceptions that, ultimately, may appear to be exclusively visual (Pazzaglia et al., 2016). The mechanism through which the visual and motor system is connected to higher-order olfactory perception is worth investigating, and could have important clinical applications. Olfaction is probably interacting with vision in multiple pathways. Olfactory sources for building integrated multisensory perceptions can develop very rapidly for perceiving and interacting with objects and actions. The actions triggered by the smelling source indicate that olfactory cues may implicitly predict and trigger action planning (Pazzaglia, 2013; Pazzaglia and Galli, 2015). These interesting possibilities await further research before this could become a useful tool in clinics. Consequently, by exploring how a greater use of odorous stimuli contributes to visual and motor planning remains a fundamental application in a clinical perspective (Galli et al., 2015; Galli and Pazzaglia, 2015; Pazzaglia and Molinari, 2016). The functional gain derived from odor integration might optimize rehabilitation programmes in case of generalized motor impairment and disturbed human social interaction.

## Author contributions

All authors listed, have made substantial, direct and intellectual contribution to the work, and approved it for publication.

### Conflict of interest statement

The authors declare that the research was conducted in the absence of any commercial or financial relationships that could be construed as a potential conflict of interest.
